# Glucose Transporter 1 Promotes the Malignant Phenotype of Non-Small Cell Lung Cancer through Integrin β1/Src/FAK Signaling

**DOI:** 10.7150/jca.30772

**Published:** 2019-08-27

**Authors:** Huanyu Zhao, Jian Sun, Jianshuang Shao, Zifang Zou, Xueshan Qiu, Enhua Wang, Guangping Wu

**Affiliations:** 1 Department of Pathology, The First Affiliated Hospital and College of Basic Medical Sciences, China Medical University, No. 155 Nanjing North Street, Heping District, Shenyang, 110001, China; 2 Guangzhou DaAn Clinical Laboratory Center, No. 74 Zhongshan Er Road, Guangzhou, 510000, China; 3 Department of Chest Surgery, The First Affiliated Hospital, China Medical University, No. 155 Nanjing North Street, Heping District, Shenyang, 110001, China

**Keywords:** Glucose transporter 1, non-small cell lung cancer, integrin β1, focal adhesion kinase, Src

## Abstract

**Background**: Glucose transporter 1 (GLUT1) is the main factor of Warburg effect, which is associated with poor prognosis in many tumors. However, the underlying molecular mechanism of GLUT1 in the progression of non-small cell lung cancer (NSCLC) is unclear.

**Methods**: We used quantitative real-time PCR to detect GLUT1 mRNA expression in bronchial brushing samples and performed Western Blot and biological behavior testing to check the effect of GLUT1 on NSCLC cell proliferation, migration, invasion and apoptosis.

**Results**: We found that the C(t) normalized value of GLUT1 in malignant bronchial brushing samples was significantly higher than that in benign samples (*P*<0.05). GLUT1 significantly increased the expressions of cyclin A, cyclin D1, cyclin E, cyclin dependent kinase 2 (CDK2), CDK4, CDK6 and matrix metalloproteinase 2 (MMP2), but decreased the expressions of p53 and p130 in NSCLC cells. The biological behavior testing indicated that GLUT1 enhanced NSCLC cell proliferation, invasion and migration but inhibited cell apoptosis. In addition, GLUT1 upregulated the expression of integrin β1 and promoted the phosphorylation of focal adhesion kinase (FAK, phosphorylation at Tyr576/577) and Src (Src phosphorylation at Tyr530). siRNA knock down of integrin β1 expression suppressed GLUT1 induced NSCLC cell biological behavior, as well as the phosphorylation of FAK and Src.

**Conclusion**: Taken together, our data confirms that GLUT1 promotes the malignant phenotype of NSCLC through integrin β1/Src/FAK signaling, which provides a new therapeutic target for the treatment and research of lung cancer.

## Introduction

Lung cancer is correlated with high morbidity and mortality. Early diagnosis of lung cancer can improve patient's cure rate. Bronchial biopsy is a method for diagnosing lung cancer. And bronchial brushing is an important supplement for bronchial biopsy. When biopsy cannot be performed (for example, lesions of the distal bronchus or if surface ulceration is severe), analysis of bronchial brushing cells is particularly important***.***Compared with homologous normal cells, malignant tumor cells have different metabolic pathways 1, 2. In aerobic conditions, glycolysis will consume more glucose to gain energy. It is called Warburg effect, which is associated with tumor progression 3. In addition to Warburg effect, glucose transporter (GLUT) facilitates glucose uptake on plasma membrane. Glucose uptake is important for tumorigenesis 4. Different members in the GLUT family are confirmed to be upregulated in malignant tumor. GLUT1 is the main factor of Warburg effect, which is the main carrier of glucose transmembrane transport 5. GLUT1 overexpression is associated with poor prognosis in many types of tumors, including lung cancer 6, 7, 8. This brings us to the idea that GLUT1 might take part in the progression of NSCLC.

MMPs are the most important enzyme for degradation of extracellular matrix (ECM). As a member of MMPs, MMP2 is widely regarded as a marker of tumor invasion and metastasis. It could significantly hydrolyze collagen I, which is the most important component of basement membrane 9, 10. In rhabdomyosarcoma cell line, GLUT1 regulates the expression of MMP2, as well as the interaction of p53 with the MMP2 promoter 11. However, it is not clear that whether there is an association between GLUT1 and MMP2 in NSCLC.

As a family of cell surface receptors, integrin is a heterodimeric transmembrane receptor formed by non-covalently bound α and β subunits. It mediated adhesion to the ECM 12. Focal adhesion kinase (FAK) is a non-receptor kinase that controls the migration, proliferation, and survival of cells 13-15. FAK can be activated by the interaction between ECM and integrin 16. GLUT1 knockdown inhibited cell proliferation, invasion, and migration through integrin β1/Src/FAK signaling pathways in breast cancer 17. We have performed RT-PCR to test GLUT1 expression and found that it was overexpressed in bronchial brushing samples of NSCLC patients 18. However, it is not clear whether GLUT1 is correlated with the progression of NSCLC.

In this study, we used quantitative real-time PCR to detect GLUT1 expression in bronchial brushing samples and performed biological behavior testing to check the effect of GLUT1 on NSCLC cell proliferation, migration, invasion and apoptosis so as to reveal its clinical value in the early diagnosis and treatment of lung cancer.

## Materials and methods

### Patients and specimens

We collected 104 bronchial brushing samples from the patients who did not underwent surgery in the First Affiliated Hospital of China Medical University from December 2014 to February 2015. There were 64 malignant samples (lung squamous cell carcinoma: 40 cases; lung adenocarcinoma: 24 cases) and 40 benign samples (pneumonia: 37 cases; tuberculosis: 3 cases). Cytology samples obtained from the bronchial brushing and the transbronchial needle aspiration (TBNA). All samples were obtained in accordance with Human Subject Research Protocols approved by the ethics committees of the First Affiliated Hospital of China Medical University (No.AF-SOP-07-1.0-01).

### Cell culture

The NSCLC cell lines (human lung adenocarcinoma cell line: H1299, SPC and A549; squamous cell carcinoma cell line: LK2 and SK-MES-1) and HBE (human bronchial epithelial cell line) cell. These cells were purchased from the American Type Culture Collection (Manassas, VA, USA). They were cultured in the medium of RPMI 1640 (Invitrogen, Carlsbad, CA, USA) or DMEM (Invitrogen, Carlsbad, CA, USA) including 10% fetal bovine serum (FBS). We detected GLUT1 protein expression in NSCLC cell lines and compared it with HBE (**Figure S1**). We selected A549 (human lung adenocarcinoma cell line, GLUT1 high expression) and LK2 (human squamous cell carcinoma cell line, GLUT1 low expression) for subsequent experiment.

### Western Blot

Total protein from NSCLC cell lines was extracted in lysis buffer and quantified by the method of Bradford. Extracted protein (Fifty micrograms) was separated by SDS-PAGE, and then transferred to polyvinylidene fluoride membranes. The membranes were blocked with 5% skim milk. After incubating overnight (4°C) with primary antibodies, the membranes were incubated with HRP-labeled secondary antibody for 2 hours at room temperature. Protein bands were examined with BioImaging System through ECL staining. We selected GAPDH as reference. The primary antibodies used in this study are shown in **Table S1**.

### Transfection

GLUT1 siRNA and control siRNA were purchased from Santa Cruz Biotechnology (CA, USA; catalog number: sc-35493), integrin β1 siRNA and control siRNA were purchased from Santa Cruz Biotechnology (CA, USA; catalog number: sc-35674), and GLUT1 plasmid were purchased from OriGene (Rockville, USA; catalog number: SC116011). They were transiently transfected to cell line with Lipofectamine 2000 (Invitrogen, Carlsbad, USA; catalog number: 11668030) according to the instruction of manufacturer.

### Quantitative real-time PCR

Quantitative real-time PCR was performed by GoTaq® qPCR Master Mix (Promega, USA) according to the instruction of manufacturer. We used 7900HT Fast Real-Time PCR System (Applied Biosystems). The C(t) value was normalized using β-actin. Each experiment was repeated by three times. The primers' sequences used in this study are as follows: GLUT1 Fwd 5'-CTGGCATCAACGCTGTCTTC-3', Rev 5'-GCCTATGAGGTGCAGGGTC-3'; β-actin Fwd 5'-GTCCACCTTCCAGCAGATGTG-3', Rev 5'-GCATTTGCGGTGGACGAT-3'.

### Transwell assay

We used transwell membranes (8μm pore polycarbonate membrane) in 24-well plates and applied Matrigel (BD Bioscience) to the upper surface of the membrane in each well. Cells (treating with different factor, 3 × 10^5^ cells in 100 μl serum-free medium) were cultured in the upper chamber, and the medium containing 10% FBS was added to the lower chamber. After incubation for 20h, the cells were stained with hematoxylin (Sigma). For each filter, we randomly selected 10 fields (400×magnification) under microscope to count the number of invaded cells.

### Cell scratch experiment

Cells were seeded in 6-well plates, and after being treated with each factor, each well was scratched with a 100μl pipette tip. The width of scratch was examined under the microscope at the time points of 1 hour and 24 hours at 3 separated sites.

### Apoptosis assay (flow cytometry)

After various treatments, cells were harvested and washed twice with phosphate‑buffered saline (PBS) by gentle shaking. Binding buffer (500μl) was added to resuspend cells, next Annexin V-FITC (5μl) and Propidium Iodide (10μl) were added to cell suspension Mix, and then darkly incubated for 15 minutes at room temperature until the detection by flow cytometry (Becton-Dickinson, San Jose, CA, USA).

### Colony formation assay

Cells (treating with different factors) were seeded in 6 cm dishes (1000 cells/dish) and cultured for 12 days. The plates were washed with PBS and stained with Giemsa. We counted the number of colonies (each with more than 50 cells) by microscopic Q.Z.D.

### Statistical analysis

We performed SPSS 17.0 for all of above analyses. All datas were reported as mean±SD, which was analyzed based on the Student's *t*-test. A two-tailed *P*<0.05 was considered to be statistically significant.

## Results

### GLUT1 mRNA expression in bronchial brushing samples

We used quantitative real-time PCR to detect bronchial brushing samples and made the standard curve for GLUT1 (target gene) and β-actin (reference gene) (**Figure S2**). GLUT1 C(t) normalization values (mean ± SD) in benign group and malignant group (adenocarcinoma group and squamous cell carcinoma group) were detected (**Table 1**). GLUT1 C(t) normalized value in malignant group was significantly higher than that in benign group (*P*<0.05).

### The effect of GLUT1 to NSCLC cell proliferation and cell cycle

**Figure 1:**
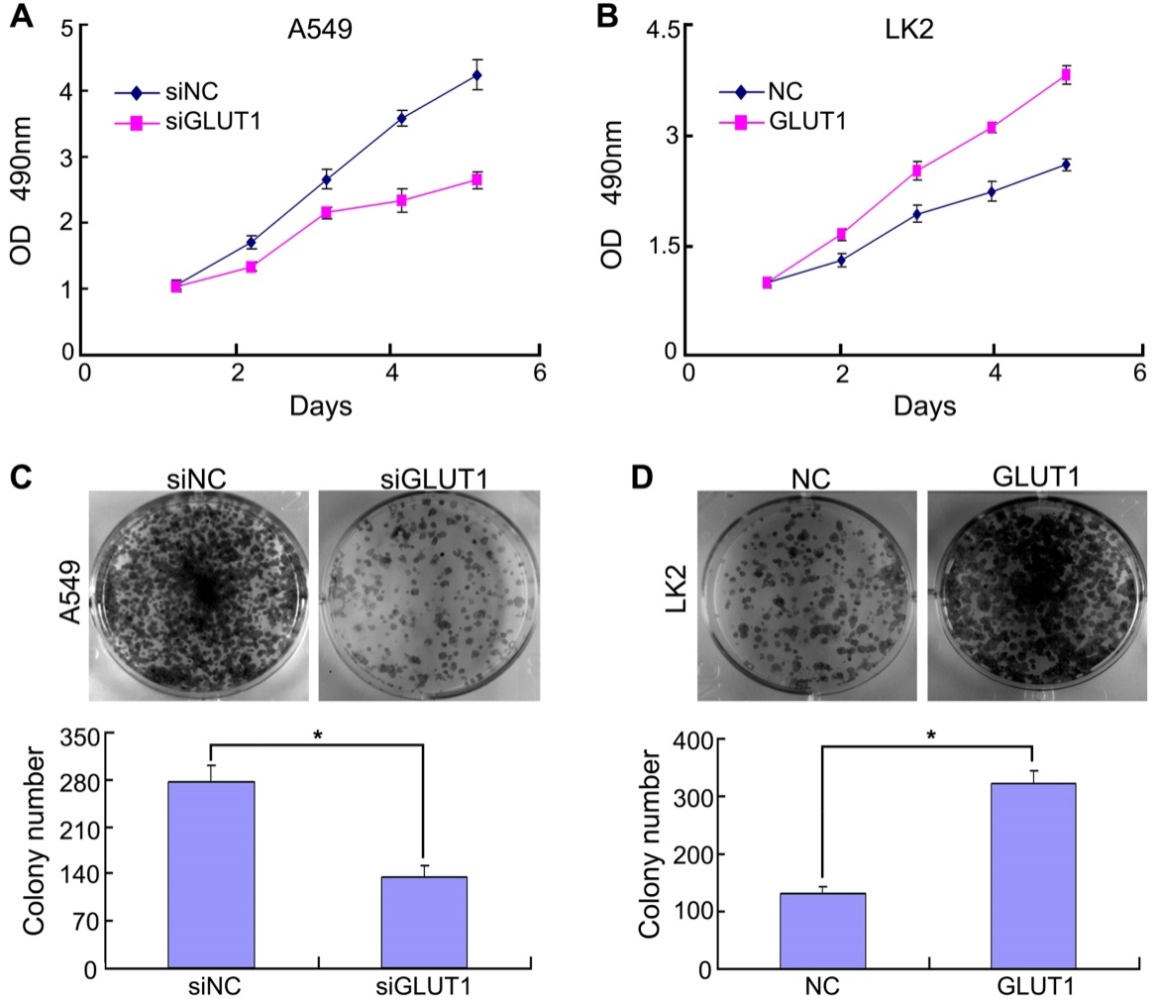
The effect of GLUT1 on NSCLC cell proliferation and colony formation. (A, B) NSCLC cell proliferation; (C, D) NSCLC cell colony formation. Adding siRNA-GLUT1 (siGLUT1) to A549 cell and GLUT1 expression plasmid (GLUT1) to LK2 cell; the graph in (C) and (D) shows the number of colony formation under different treatments; ^*^, *P* <0.05 compared with negative control; NC, negative control; si-NC, scramble siRNA for negative control; Error bars, S.D.

Previous study confirmed that Ghrelin enhanced GLUT1 expression and promoted oral cancer cell proliferation 19. GLUT1 overexpression promoted cell viability and proliferation 20. Above reports reminded us that there might be a relationship between GLUT1 and NSCLC cell proliferation. Based on GLUT1 protein expression in NSCLC cell lines (**Figure S1**), we selected A549 (GLUT1 high expression) and LK2 (GLUT1 low expression) for subsequent experiment. We transfected siRNA-GLUT1 to A549 and GLUT1 expression plasmid to LK2 cell line. The result of MTT and colony formation assays showed that siRNA-GLUT1 significantly inhibited cell colony formation and proliferation in A549 cell and GLUT1 transfection had the opposite effect in LK2 cell (*P*<*0.05*, **Figure 1**).

**Figure 2:**
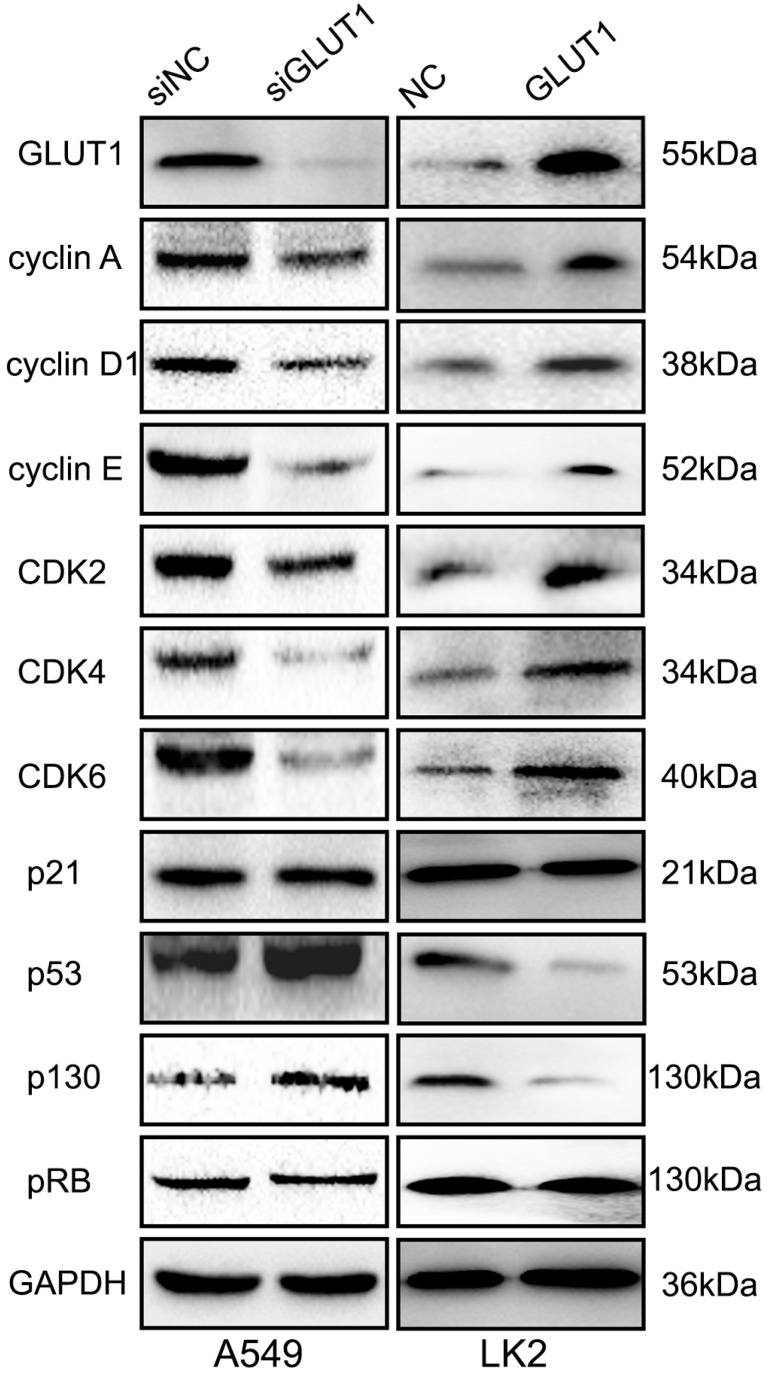
The effect of GLUT1 on the cell cycle correlated proteins in NSCLC cells. We added siRNA-GLUT1 (siGLUT1) to A549 cell and GLUT1 expression plasmid (GLUT1) to LK2 cell; NC, negative control; si-NC, scramble siRNA for negative control.

For detecting the effect of GLUT1 to cell cycle, we performed Western Blot to test the cell cycle correlated proteins (cyclin A, cyclin D1, cyclin E, CDK2, CDK4, CDK6, p21, p53, pRB, p130). The result showed that adding siRNA-GLUT1 to A549 cell significantly decreased the expressions of cyclin A, cyclin D1, cyclin E, CDK2, CDK4, CDK6 and increased the expressions of p53 and p130, but had no effect on p21 and pRB (**Figure 2**). GLUT1 transfection had the opposite effect in LK2 cell. These results indicated that GLUT1 could regulated cell cycle of NSCLC cells.

### The effect of GLUT1 to cell migration of NSCLC cell

**Figure 3:**
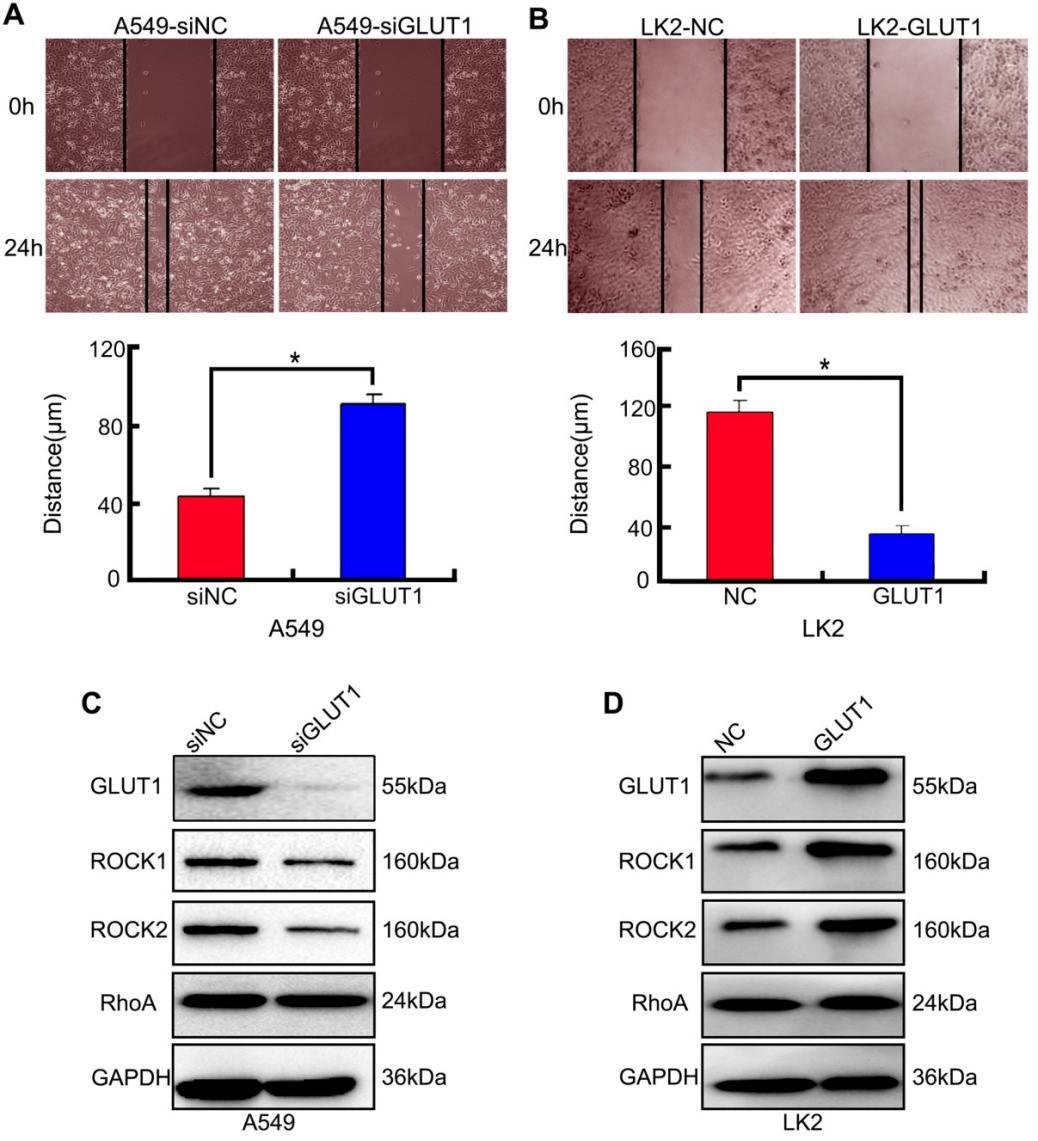
The effect of GLUT1 on NSCLC cell migration and correlated proteins. (A, B) NSCLC cell migration; (C, D) the expressions of ROCK1, ROCK2 and RhoA. We added siRNA-GLUT1 (siGLUT1) to A549 cell and GLUT1 expression plasmid (GLUT1) to LK2 cell; the graph in (A) and (B) shows the distance of cell migration under different treatments; ^*^, *P*<0.05 compared with negative control; NC, negative control; si-NC, scramble siRNA for negative control; Error bars, S.D.

We used cell scratch to detect the effect of siRNA-GLUT1 and GLUT expression plasmid to on NSCLC cell migration. The results showed that siRNA-GLUT1 inhibited cell migration in A549 cell and GLUT1 transfection had the opposite effect in LK2 cell (*P*<0.05, **Figure 3A and 3B**).

The result of Western Blot showed that siRNA-GLUT1 significantly inhibited the expression of Rho-associated coiled-coil containing kinase 1 (ROCK1) and ROCK2 in A549 cell, and GLUT1 had the opposite effect in LK2 cell. However, siRNA-GLUT1 or GLUT1 transfection had no effect on RhoA expression (**Figure 3C and 3D**).

### The effect of GLUT1 to cell invasion of NSCLC cell

**Figure 4:**
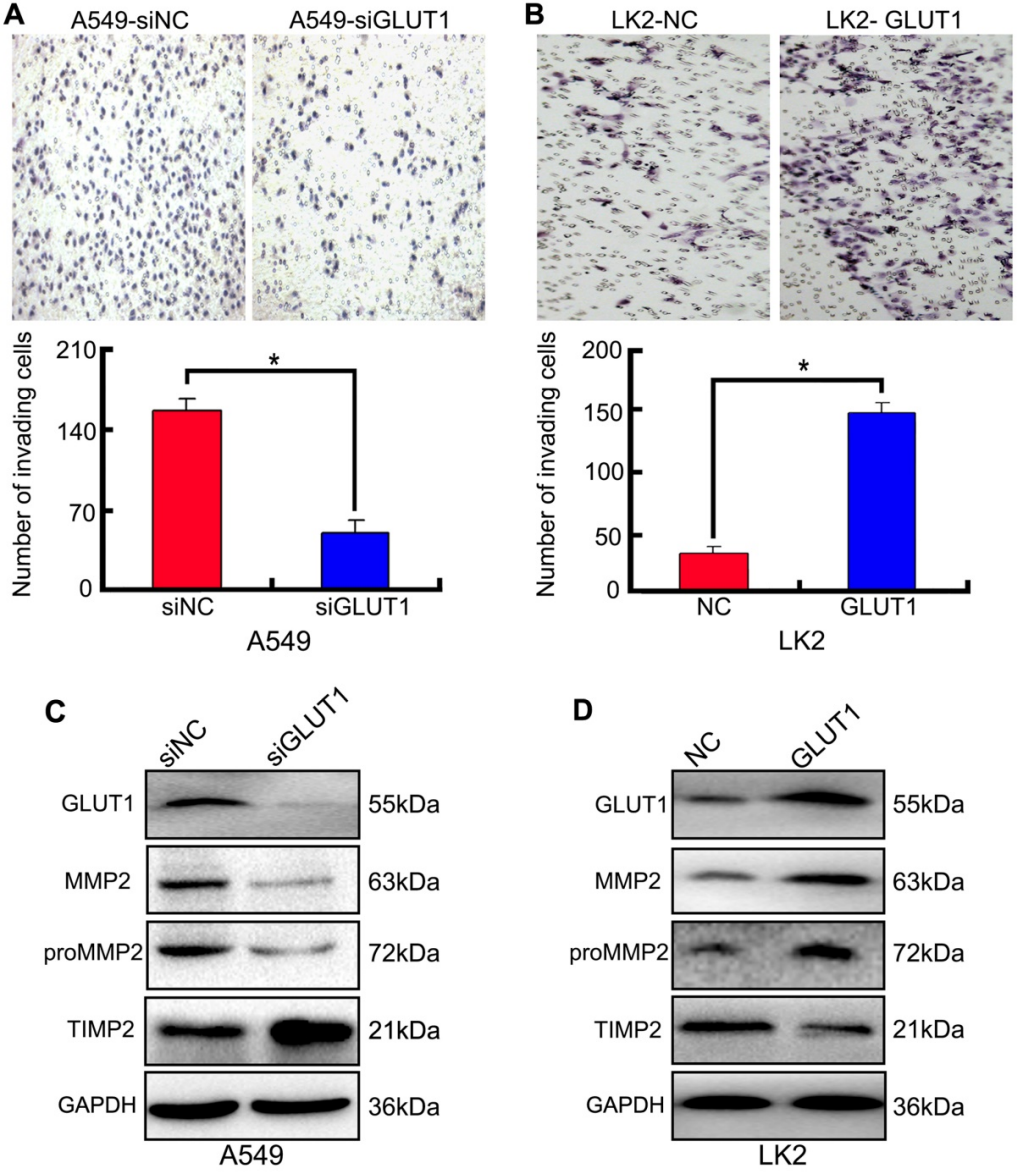
The effect of GLUT1 on NSCLC cell invasion and correlated proteins. (A, B) NSCLC cell invasion; (C, D) the expressions of MMP2, proMMP2 and TIMP2. We added siRNA-GLUT1 (siGLUT1) to A549 cell and GLUT1 expression plasmid (GLUT1) to LK2 cell; the graph in (A) and (B) shows the number of invading cells under different treatments; ^*^, *P*<0.05 compared with negative control; NC, negative control; si-NC, scramble siRNA for negative control; Error bars, S.D.

We used Transwell to detect the effect of siRNA-GLUT1 and GLUT1 expression plasmid on NSCLC cell invasion. The results showed that adding siRNA-GLUT1 inhibited cell invasion in A549 cell and GLUT1 transfection had the opposite effect in LK2 (*P*<0.05, **Figure 4A and 4B**).

Tissue inhibitor of matrix metalloproteinase 2 (TIMP2) is a inhibitor of MMP2. The balance between MMP2 and TIMP2 plays a key role in tumor cell invasion and metastasis 21. proMMP2/MMP2 activation pathway is critical for cancer cell metastasis 22. The result of Western Blot showed that adding siRNA-GLUT1 significantly inhibited the expression of MMP2 and proMMP2 in A549 cell, and GLUT1 transfection had the opposite effect in LK2 cell. The result indicated that GLUT1 had a positive effect on the expressions of MMP2 and proMMP2 in NSCLC cells. Meanwhile, GLUT1 had negative effect on TIMP2 expression (**Figure 4C and 4D**).

### The effect of GLUT1 to NSCLC cell apoptosis

**Figure 5:**
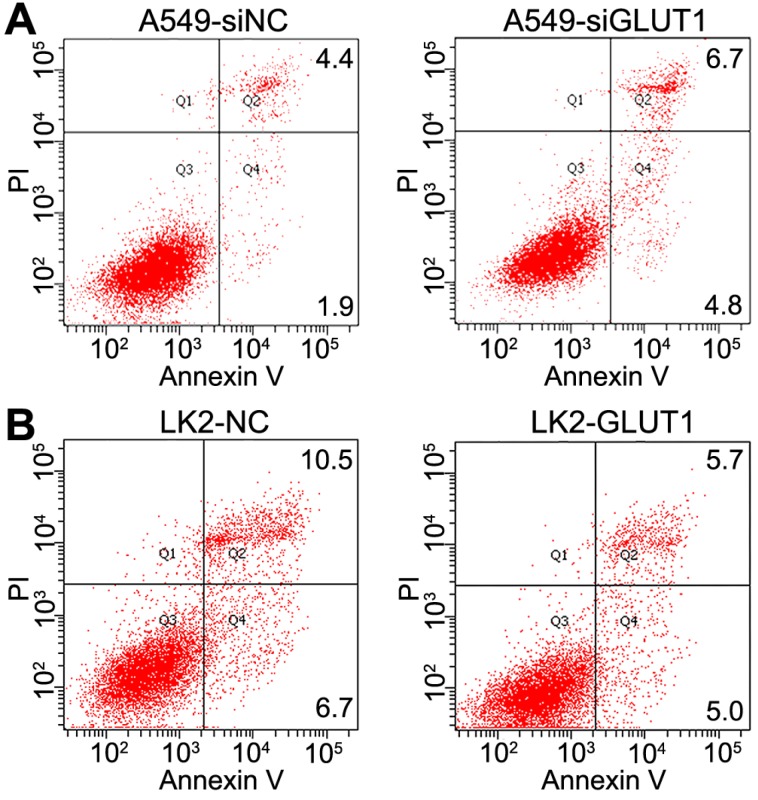
The effect of GLUT1 on the apoptosis in A549 and LK2 cells. We added siRNA-GLUT1 (siGLUT1) to A549 cell (A) and GLUT1 expression plasmid (GLUT1) to LK2 cell (B); NC, negative control; si-NC, scramble siRNA for negative control.

We transiently transfected siRNA-GLUT1 and GLUT1 expression plasmid to NSCLC cells. Annexin V staining assay was used to determine the effect of GLUT1 on apoptosis of A549 and LK2 cells. The apoptosis rate was markedly increased after siRNA-GLUT1 transfection in A549 cell, but significantly decreased after GLUT1 transfection in LK2 cell (*P*<0.05, **Figure 5A and 5B**). The results indicated that GLUT1 could inhibit the apoptosis of NSCLC cells.

### GLUT1 regulates NSCLC cell proliferation, migration, invasion and apoptosis through integrin β1/Src/FAK signaling pathway

Integrin could regulate many biological processes, including cell migration and proliferation 23-25. Integrin has not catalytic activity, but it can bind and activate signaling proteins, such as non-receptor tyrosine kinase, including FAK and Src 26. The association between FAK and integrin β1 is required for FAK activation 27.

**Figure 6:**
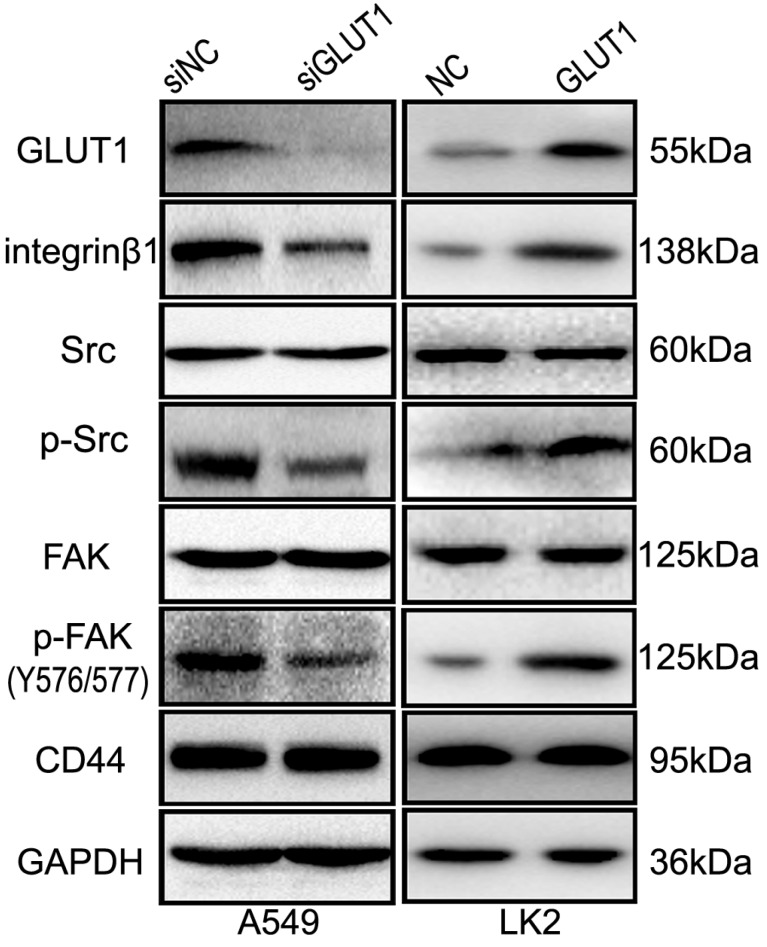
The effect of GLUT1 on integrin β1/Src/FAK signaling in NSCLC cells. We added siRNA-GLUT1 (siGLUT1) to A549 cell and GLUT1 expression plasmid (GLUT1) to LK2 cell; NC, negative control; si-NC, scramble siRNA for negative control.

We used Western Blot to detect the effect of GLUT1 on integrin β1, FAK and Src. The result showed that GLUT1 upregulated the expression of integrin β1 and promoted the phosphorylation of FAK (Tyr576/577 phosphorylated) and Src (Tyr530 phosphorylated), but there is no effect on CD44 expression (**Figure 6**). After co-transfection of GLUT1 expression plasmid with the siRNA-integrin β1, the effect of GLUT1 on cell proliferation, migration, invasion and apoptosis was significantly suppressed (**Figure S3**), as well as the phosphorylation of FAK (Tyr576/577 phosphorylated) and Src (Tyr530 phosphorylated) (**Figure S4**).

Above results indicate that GLUT1 may regulate the proliferation, migration, invasion and apoptosis of NSCLC cell through integrin β1/Src/FAK signaling.

## Discussion

As a member of GLUT family, GLUT1 plays an important role in Warburg effect 28. GLUT1 overexpression is associated with the prognosis of malignant tumor patients 29, 30. GLUT1 expression is significantly correlated with MMP2 expression in tumor cells 11. We found that GLUT1 was overexpressed in bronchial brushing samples of NSCLC patients, and GLUT1 expression in NSCLC tissues was higher than that in paracancerous tissues 31. However, the potential molecular mechanism that GLUT1 regulates NSCLC cell invasion, proliferation and migration is still unclear.

We have used RT-PCR to detect GLUT1 mRNA expression of bronchial brushing specimens, which might be a useful adjunct to cytology diagnosis. It was more sensitive than cytology but its lower specificity 18. In this study, we used quantitative real-time PCR to detect bronchial brushing samples. This method could quantitatively detect the samples, which is better than RT-PCR. Consistent with previous results, GLUT1 mRNA expression in malignant group is significantly higher than that in benign group. It has been reported that Ghrelin modulates GLUT1 expression so as to enhance oral cancer cell proliferation indirectly 19. GLUT1 overexpression promoted cell viability and colony formation of neck squamous cell carcinoma 20. Gene promoter methylation is associated with high-grade tumor 32, and GLUT1 promotor methylation was related with survival rate of tumor cells 33. It presents us with a hypothesis that GLUT1 might have an effect on NSCLC cell proliferation. We transfected siRNA-GLUT1 to A549 cell and found that cell proliferation and colony formation was significantly inhibited (*P*<0.05), as well as the expressions of cyclin D1, cyclin E, CDK2, CDK4 and CDK6, but the expressions of p53 and p130 were enhanced, as well as cell apoptosis. GLUT1 transfection to LK2 cell had the opposite effect. The phases of the cell cycle are regulated by many factors. The G1 phase is mainly mediated by CDK2-cyclin E and CDK4/6-cyclin D1 complexes and the G1/S phase transition is influenced by CDK2-cyclin A complex 34-37. CDK2-cyclin E and CDK4/6-cyclin D1 complexes are important for the promotion of G1/S checkpoint transition in cell cycle 38, 39. The transcription factor p53 is a tumor suppressor, regulating many target genes 40. p130 is involved in cellular senescence 41. Cellular senescence means that cell proliferation is suppressed 42. RB phosphorylation is important for the G1 to S phase transition 43. The cyclin-dependent kinase inhibitor p21 promotes cell cycle arrest by many stimuli 44. As a member of the cell adhesion molecule family, CD44 plays a crucial role in tumor growth and motility 45. Based on the above reports and our results, we concluded that GLUT1 regulated NSCLC cell proliferation through cyclin D1, cyclin E, CDK2, CDK4, CDK6, p53 and p130.

It is well known that MMP2 is a marker of tumor invasion and metastasis. GLUT1 could regulate the expression of MMP2 11. In this study, we found that GLUT1 could significantly enhance cell migration and invasion in NSCLC cell lines, and GLUT1 could upregulate the MMP2 expression. The detailed molecular mechanism of MMP2 mediated by GLUT1 need to do further studies.

ROCK is one of the most important effector molecules in the downstream of Rho signaling 46. ROCK proteins (ROCK1 and ROCK2) are activated by a small GTPase protein RhoA. It has been reported that RhoA/ROCK signaling pathway is involved in regulating actin cytoskeleton and promoting actin globulin contraction 47, 48. In this study, GLUT1 could increase the expressions of ROCK1 and ROCK2, but have not effect on RhoA. So the regulation of the NSCLC cell migration by GLUT1 might not be mediated by RhoA/ROCK signaling.

Integrins are cell surface transmembrane receptors that identify and interact with ECM proteins, playing important roles in activating signaling pathways related to cytoskeleton 49. Focal adhesion kinase (FAK) is a non-receptor kinase that takes part in cell migration and proliferation 13-15. The interaction of FAK with integrin β1 is required for FAK activation 27. Integrin-linked FAK exhibited tyrosine phosphorylation and significantly associated with Src through their SH2 domains 50. Src is a critical mediator of FAK-regulated migratory and invasive activity 51. In NSCLC cell, GLUT1 could upregulate the expressions of integrin β1, Src and FAK, as well as p-Src (Tyr 530 phosphorylated) and p-FAK (Tyr576/577 phosphorylated). These results showed that phosphorylated Src and FAK was important for the regulation of the NSCLC cell migration and invasion by GLUT1. Above results indicate that GLUT1 may regulate the progression of NSCLC through integrin β1/Src/FAK signaling.

Integrin β1 has been reported to play an important role in regulating cell migration, proliferation and invasion, which could activate down-stream FAK/Src signaling 52-54. So we co-transfected GLUT1 expression plasmid with the siRNA-integrin β1 to LK2 cell. The results showed that adding siRNA-integrin β1 suppressed the effect of GLUT1 on cell proliferation, invasion, migration, and apoptosis. The GLUT1 induced phosphorylation of FAK (Tyr576/577 phosphorylated) and Src (Tyr530 phosphorylated) was significantly inhibited by siRNA-integrin β1. We could conclude that GLUT1 regulates the cell biological behavior through integrin β1.

The genetic variation of GLUT1 could be used in predicting survival of patients with NSCLC in early stage 55. The association between positron emission tomography (PET) parameters and GLUT1 expression is an important symbol for judging prognosis in lung adenocarcinoma 56. This is an indication that GLUT1 expression has great clinical significance for NSCLC patients. In this study, we confirm that GLUT1 mRNA expression in bronchial brushing samples of NSCLC is significantly higher than that in benign group and GLUT1 promotes the malignant phenotype of NSCLC through integrin β1/Src/FAK signaling, which provides a new target for the research and treatment of lung cancer.

## Supplementary Material

Supplementary figures and table.

## Figures and Tables

**Table 1:** The C(t) normalization value (mean ± SD) of GLUT1 in bronchial brushing samples.

Group	Benign (n=40)	Malignant (n=64)	SCC (n=40)	ADE (n=24)
GLUT1 C(t) normalization value	0.15±0.03	0.85±0.19*	1.18±0.29*	0.31±0.07*

*, *P*<0.05 compared with benign group; SCC, squamous cell carcinoma; ADE, adenocarcinoma.

## Abbreviations

GLUT1: glucose transporter 1
MMP: matrix metalloproteinase
ECM: extracellular matrix
NSCLC: non-small cell lung cancer
FAK: focal adhesion kinase
TIMP2: tissue inhibitor of metalloproteinases 2
CDK: cyclin dependent kinase
ROCK: Rho-associated coiled-coil containing kinase
PBS: phosphate‑buffered saline
FBS: fetal bovine serum
