# Supplementary material for: Glucose Transporter 1 Promotes the Malignant Phenotype of Non-Small Cell Lung Cancer through Integrin β1/Src/FAK Signaling
Source: J Cancer. 2019 Aug 27;10(20):4989–97. doi: 10.7150/jca.30772 (PMC6775508; doi:10.7150/jca.30772)
Supplement: Supplementary file 1 — Supplementary figures and table. [file jcav10p4989s1.pdf]

Supplementary figures and tables

Table S1. The antibodies used in this study.

Figure S1. The expression of GLUT1 in NSCLC cell lines.

Figure S2. The standard curve for β-actin and GLUT1. (A) the standard curve for β-actin (reference gene), the standard curve equation: Y = -3.2275 × LOG (X0) + 28.731, X=LOG (X0) correlation coefficient: 0.9884; amplification efficiency： 104.1%. (B) the standard curve for GLUT1 (target gene), the standard curve equation: Y= -3.1573 × LOG (X0) + 32.881, X=LOG (X0); correlation coefficient: 0.9803; amplification efficiency: 107.4%. LOG: logarithms; CT: cycle threshold.

Figure S3. The effect of siRNA-integrin β1 on GLUT1-mediated NSCLC cell biological behavior. (A) NSCLC cell migration; (B) NSCLC cell invasion; (C) NSCLC cell colony formation; (D) NSCLC cell proliferation; (E) NSCLC cell apoptosis. We co-transfected GLUT1 expression plasmid with siRNA-integrin β1 to LK2 cell; the graph in (A) shows the distance of cell migration under different treatments; the graph in (B)-(C) shows the number of invading cells and colony formation under different treatments; a, negative control; b, tranfection of GLUT1 expression plasmid; c, co-transfection of GLUT1 expression plasmid with siRNA-integrin β1.

Figure S4. The effect of siRNA-integrin β1 on GLUT1-mediated correlated proteins in LK2 cell. The integrin β1/Src/FAK signaling correlated proteins: Src, p-Src, FAK, p-FAK, integrin β1. We co-transfected GLUT1 expression plasmid

(GLUT1) with the siRNA-integrin β1 (si-integrin β1) to LK2 cell; si-NC, scramble siRNA for negative control.

Table S1. The antibodies used in this study.

Antibody	Vendor	Catalog number	Uses*
GLUT1	Santa Cruz Biotechnology	sc-377228	WB (1: 400)
cyclin E	Santa Cruz Biotechnology	sc-247	WB (1: 300)
cyclin A	Santa Cruz Biotechnology	sc-239	WB (1: 300)
cyclin D1	Santa Cruz Biotechnology	sc-20044	WB (1: 300)
MMP2	Santa Cruz Biotechnology	sc-13594	WB (1: 300)
TIMP2	Santa Cruz Biotechnology	sc-21735	WB (1: 400)
CDK2	Santa Cruz Biotechnology	sc-6248	WB (1: 200)
CDK4	Santa Cruz Biotechnology	sc-23896	WB (1: 400)
CDK6	Santa Cruz Biotechnology	sc-7961	WB (1: 400)
p53	Santa Cruz Biotechnology	sc-126	WB (1: 300)
p21	Santa Cruz Biotechnology	sc-6246	WB (1: 300)
p130	Santa Cruz Biotechnology	sc-53641	WB (1: 300)
Rb	Santa Cruz Biotechnology	sc-102	WB (1: 300)
Phospho-Rb	Santa Cruz Biotechnology	sc-271930	WB (1: 300)
integrin β1	Santa Cruz Biotechnology	sc-13590	WB (1: 400)
FAK	Cell Signaling Technology	3285	WB (1: 1000)
Phospho-FAK	Cell Signaling Technology	3281	WB (1: 1000)
Src	Santa Cruz Biotechnology	sc-130124	WB (1: 300)
Phospho-Src	Santa Cruz Biotechnology	sc-136012	WB (1: 300)
ROCK1	Santa Cruz Biotechnology	sc-17794	WB (1: 300)

CD44	Santa Cruz Biotechnology	sc-9960	WB (1: 300)
GADPH	Santa Cruz Biotechnology	sc-32233	WB (1: 1000)
ROCK2	Santa Cruz Biotechnology	sc-398519	WB (1: 300)
RhoA	Santa Cruz Biotechnology	sc-418	WB (1: 300)

*WB, Western Blot. Antibody dilutions are indicated in bracket.

Figure S1

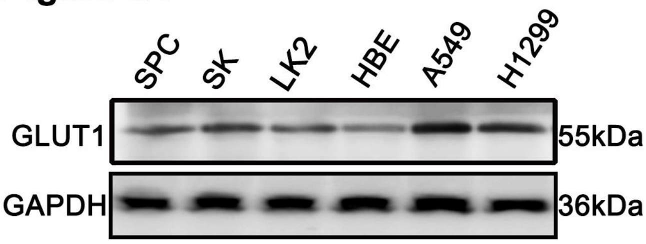

Figure S2

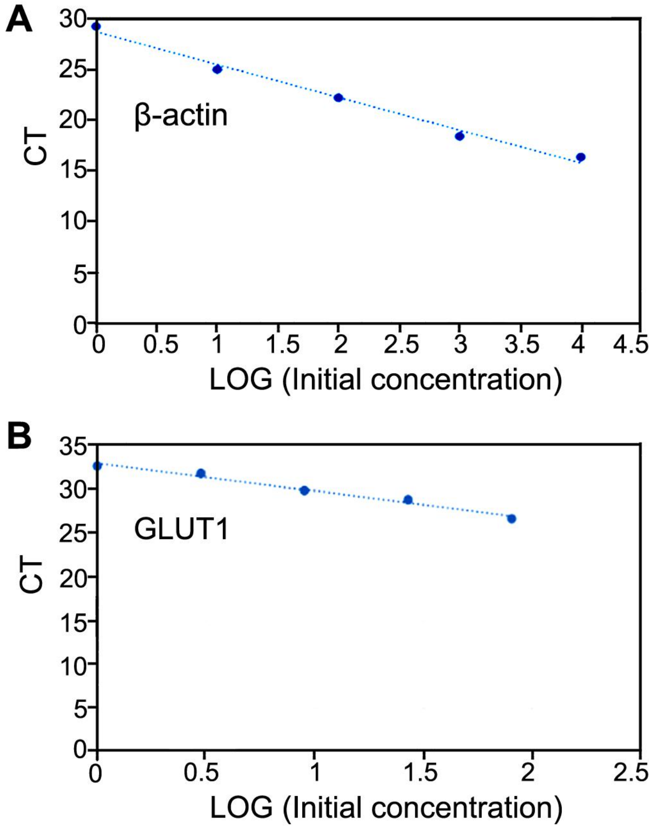
A
30
25
20
15
10
5
0
CT
β-actin
0
0.5
1
1.5
2
2.5
3
3.5
4
4.5
LOG (Initial concentration)
B
35
30
25
20
15
10
5
0
CT
GLUT1
0
0.5
1
1.5
2
2.5
LOG (Initial concentration)

Figure S3

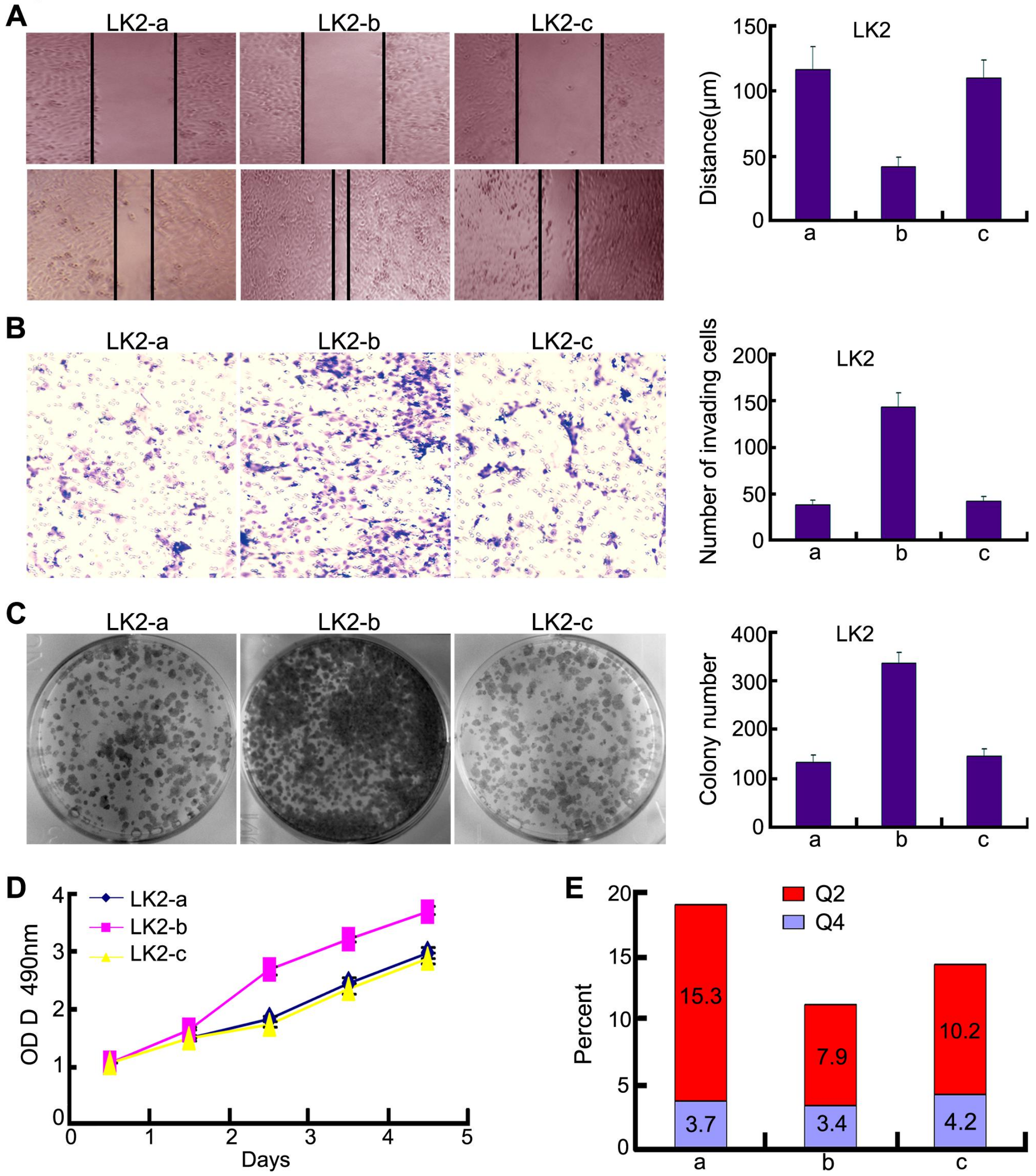
A
LK2-a
LK2-b
LK2-c
LK2
Distance(μm)
150
100
50
0
a
b
c
B
LK2-a
LK2-b
LK2-c
LK2
Number of invading cells
200
150
100
50
0
a
b
c
C
LK2-a
LK2-b
LK2-c
LK2
Colony number
400
300
200
100
0
a
b
c
D
LK2-a
LK2-b
LK2-c
OD D 490nm
4
3
2
1
0
0
1
2
3
4
5
Days
E
Q2
Q4
Percent
20
15
10
5
0
15.3
3.7
7.9
3.4
10.2
4.2
a
b
c

Figure S4

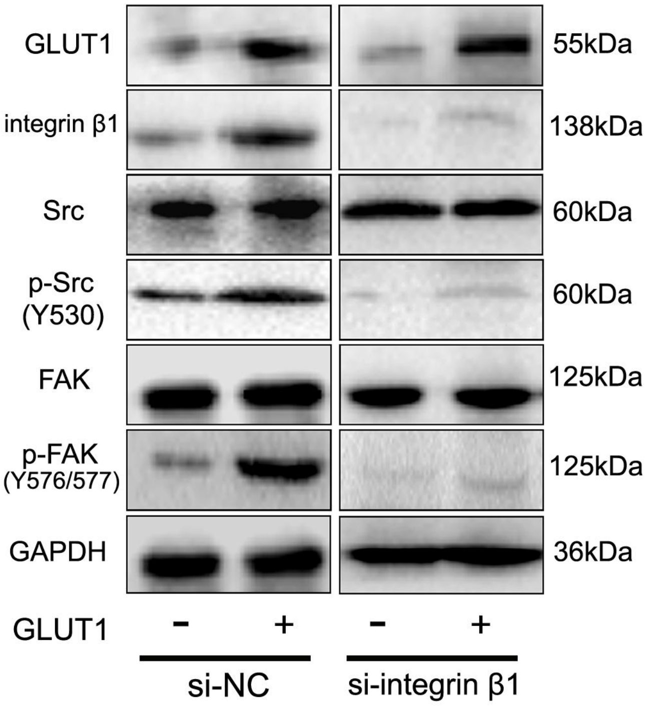

GLUT1
integrin β1
Src
p-Src (Y530)
FAK
p-FAK (Y576/577)
GAPDH
55kDa
138kDa
60kDa
60kDa
125kDa
125kDa
36kDa
GLUT1
-
+
-
+
si-NC
si-integrin β1
